# Epidemiologie und Therapie der behandlungsbedürftigen Frühgeborenenretinopathie. Die Hannoveraner Daten im Retina.net ROP-Register von 2001 bis 2017

**DOI:** 10.1007/s00347-021-01528-9

**Published:** 2021-11-22

**Authors:** Stella H. Akman, Johanna M. Pfeil, Andreas Stahl, Stephanie Ehlers, Carolin Böhne, Bettina Bohnhorst, Carsten Framme, Dorothee Brockmann, Anna Bajor, Christina Jacobsen, Karsten Hufendiek, Amelie Pielen, Retina.net ROP Register-Studiengruppe, Stella H. Akman, Anna Bajor, Julia Behrens, Dorothee Brockmann, Carsten Framme, Karsten Hufendiek, Christina Jacobsen, Benjamin Luger, Sophia Mies, Amelie Pielen, Nora Wojtera, Bettina Bohnhorst, Carolin Böhne, Stephanie Ehlers, Carina Zirkler

**Affiliations:** 1 Universitätsklinik für Augenheilkunde, Medizinische Hochschule Hannover, Carl-Neuberg-Str. 1, 30625 Hannover, Deutschland; 2 Klinik und Poliklinik für Augenheilkunde, Universitätsmedizin Greifswald, Ferdinand Sauerbruch Str., 17475 Greifswald, Deutschland; 3 Klinik für Pädiatrische Pneumologie, Allergologie und Neonatologie, Medizinische Hochschule Hannover, Carl-Neuberg-Str. 1, 30625 Hannover, Deutschland

**Keywords:** Intravitreale Injection, Anti-VEGF, Laserkoagulation, Nonatologische Komorbidität, Bronchopulmonale Dysplasie, Intravitreal injection, Anti-VEGF, Laser coagulation, Neonatal comorbidy, Bronchopulmonary dysplasia

## Abstract

**Hintergrund:**

Das Retina.net ROP-Register erhebt Daten von Kindern, die eine seltene behandlungsbedürftige Frühgeborenenretinopathie (Retinopathy of Prematurity, ROP) entwickeln. *Ziel* dieser Auswertung ist die Untersuchung der Daten zur behandlungsbedürftigen ROP, Epidemiologie, Therapie und deren Änderungen über einen Zeitraum von 15 Jahren an der Medizinischen Hochschule Hannover.

**Methoden:**

Analyse der Daten der therapiebedürftigen Fälle der ROP eines Zentrums für die Geburtsjahre 2001 bis 2016 (Therapie in 2002 bis 2017) als Gesamtzeitraum und in 5 Abschnitten.

**Ergebnisse:**

Es wurden 65 Kinder behandelt (23 weiblich), davon wurden 11 (16,9 %) extern auf ROP gescreent und zur ROP-Behandlung zugewiesen. Für den Zeitraum 2006 bis 2016 lag die Inzidenz der behandlungsbedürftigen ROP unter den gescreenten Kindern bei 4,1 %. Das mittlere Gestationsalter betrug 25,7 Schwangerschaftswochen (SSW) (Standardabweichung [SA] = 1,8), das Geburtsgewicht 763 g (SA = 235), das postmenstruelle Alter bei Behandlung 38,2 Wochen (SA = 3,2), das postnatale Alter 12,4 Wochen (SA = 3,2). Über die Zeit zeigte sich kein signifikanter Unterschied der demografischen Parameter. Am häufigsten (57 Augen bei 31 Kindern) wurde eine Zone II, 3+-Erkrankung behandelt; 58 Kinder erhielten eine Laserkoagulation (*N* = 114 Augen), 7 Kinder bilateral eine Anti-VEGF-Therapie (Bevacizumab) (*N* = 14 Augen), welche ab 2014 eingesetzt wurde. Eine Wiederbehandlung bei Wiederaufflammen der behandlungsbedürftigen ROP war in einem Fall nach initialer Laserbehandlung notwendig. Kinder mit behandlungsbedürftiger ROP zeigten häufig neonatologische Komorbiditäten, in mehr als 90 % Beatmung, bronchopulmonale Dysplasie und erhielten Transfusionen.

**Schlussfolgerung:**

Dies ist die erste monozentrische Auswertung über 15 Jahre im Rahmen des Retina.net ROP-Registers. Ab 2014 sehen wir einen Wechsel von der Laserkoagulation zur Anti-VEGF-Therapie (Bevacizumab), während im betrachteten Kollektiv die demografischen Daten und Behandlungsparameter weitgehend konstant waren.

## Abstract (en)

**Background:**

The Retina.net ROP registry documents data of preterm infants developing stages of retinopathy of prematurity (ROP) that need ROP treatment. The aim of this analysis was to investigate data regarding epidemiology, therapy and changes over time (15 years) in a single participating center (Hannover Medical School, MHH).

**Methods:**

Analysis of data of infants treated for ROP at a single center over time (birth 2001–2016, ROP treatment in 2002–2017).

**Results:**

Overall, 65 infants were treated (23 female). In 11 infants (16.9%) ROP screening was conducted externally and infants were transferred to the MHH for ROP treatment. Between 2006 and 2016, incidence of ROP requiring treatment among infants screened for the development of ROP was 4.1%. Mean gestational age was 25.7 weeks (standard deviation, SD 1.8), mean birth weight 763 g (SD 235), postmenstrual age at treatment 38.2 weeks (SD 3.2), postnatal age 12.4 weeks (SD 3.2). There was no significant change in parameters over time. ROP zone II, stage 3+ was most frequently treated (57 eyes of 31 infants). 58 infants were treated with laser (114 eyes), 7 infants were treated with anti-VEGF (bevacizumab, bilateral, 14 eyes) from 2014 onwards. Retreatment due to recurrence of ROP was necessary in one infant after initial laser coagulation. Infants with ROP requiring treatment often presented with neonatal comorbidities, ventilation in more than 90%, bronchopulmonary dysplasia, and received transfusions.

**Conclusion:**

This is the first monocentric analysis over 15 years originating from the Retina.net ROP registry. In this cohort we see a change in ROP therapy from laser coagulation to anti-VEGF (bevacizumab) from 2014 onwards, demographic data and treatment parameters remained relatively stable over time.

## Hintergrund und Fragestellung

In Deutschland werden nur sehr wenige Kinder mit einer Frühgeborenenretinopathie (englisch: „retinopathy of prematurity“ [ROP]) behandlungsbedürftig [7, 8, 18]. Im Jahr 2012 wurde das Retina.net ROP-Register ins Leben gerufen, um Daten von möglichst vielen Verläufen bei behandlungsbedürftiger ROP aus ganz Deutschland in einer gemeinsamen Datenbank zu erfassen [16]. Eine Teilnahme an der Datenerhebung steht allen deutschen Zentren offen, die Kinder mit einer ROP behandeln. Derzeit nehmen 19 Zentren am ROP-Register teil, die insgesamt Datensätze von mehr als 380 behandelten Kindern gesammelt haben [19]. Für den Zeitraum von 2011 bis 2015 wurden Daten von 150 Kindern (292 Augen) aus ganz Deutschland ausgewertet und publiziert, dies entspricht ca. 10–15 % der in diesem Zeitraum behandelten Frühgeborenen in Deutschland [19]. Die Medizinische Hochschule Hannover (MHH) nimmt seit 2014 am Retina.net ROP-Register teil und nutzt die Datenbank, um alle Kinder, die aufgrund einer ROP an der MHH behandelt werden, systematisch zu erfassen. Das Ziel der vorliegenden Auswertung ist zu überprüfen, ob sich demografische Parameter (Gestationsalter [GA], Geburtsgewicht, Alter bei Behandlung) sowie Parameter zur Behandlung selbst (Art der Behandlung) über einen Zeitraum von 15 Jahren an der MHH geändert haben. Außerdem sollen die lokalen Daten aus der Medizinischen Hochschule Hannover (MHH) mit den veröffentlichten Daten des Retina.net-Gesamtregisters und anderer Register verglichen werden.

## Methoden

Das Retina.net ROP-Register ist ein bundesweites Register, in dem Daten von Frühgeborenen erfasst werden, die eine behandlungsbedürftige ROP entwickeln. Es ist unter der Nummer DRKS00004522 im Deutschen Register für klinische Studien registriert (www.drks.de). Es liegen positive Voten der federführenden Ethikkommission der Universität Greifswald (Nummer BB 165/19) und aller beteiligten Ethikkommissionen der teilnehmenden Zentren vor (Ethikvotum MHH Nr. 2239-2014). Die MHH nutzt seit 2014 die Möglichkeit, ihre behandelten ROP-Fälle in der Datenbank des Retina.net ROP-Registers zu dokumentieren. Nach schriftlicher Einwilligung der Sorgeberechtigten können prospektiv Daten erfasst werden, welche pseudonymisiert gespeichert werden. Eine retrospektive Dateneingabe ist ohne Einwilligung möglich, da die Daten anonymisiert gespeichert werden. Für die aktuelle Auswertung wurde auch die Möglichkeit der retrospektiven Dateneingabe im Retina.net ROP-Register genutzt. So konnten Kinder, die ab 2002 an der MHH behandelt wurden, also vor dem eigentlichen Beginn der Teilnahme der MHH am Register, in die Auswertung mit aufgenommen werden, und dadurch konnte ein Zeitraum von 15 Jahren betrachtet werden. Es handelt sich um eine nichtinterventionelle Datenerhebung unabhängig von der Behandlung (Laser oder Anti-VEGF). In der aktuellen Auswertung wird eine Subgruppenanalyse derjenigen Kinder durchgeführt, die in den Jahren 2001 bis 2016 geboren wurden und deren ROP im Zeitraum 2002 bis 2017 in der Augenklinik der MHH behandelt wurde (*N* = 65, davon 10 prospektiv, 55 retrospektiv). Ein Teil der Daten wurde in der Gesamtauswertung des Registers für die Geburtsjahrgänge 2011 bis 2015 analysiert [18, 19]. Die neonatologischen Parameter waren im primären Registerauszug, basierend auf den Akteneinträgen der Augenklinik, nicht auswertbar, daher erfolgte eine erneute Datenerhebung in enger Kooperation mit der Neonatologie aus den elektronischen Akten und digitalisierten Kurven.

In der vorliegenden Arbeit werden demografische Daten zum Zeitpunkt der Geburt und der Behandlungsentscheidung, neonatologische Parameter sowie Behandlungsparameter im Verlauf der Zeit sowie im Vergleich zum Gesamtdatensatz untersucht. Bei kontinuierlichen Variablen werden der Mittelwert, die Standardabweichung (SA) sowie der minimale (min) und maximale (max) Wert angegeben, bei kategorialen Variablen wird die Verteilung der Kinder auf die Kategorien in Prozent dargestellt. Für Parameter, die nicht für alle Kinder bzw. Augen vorlagen, wird die zugrunde liegende Anzahl durch Angabe von *N* angezeigt. Die statistischen Analysen erfolgten unter Verwendung von SPSS V.27 (IBM Corp., Armonk, NY, USA).

## Ergebnisse

### Demografische Parameter

Im Beobachtungszeitraum wurden 65 Kinder an der MHH aufgrund einer ROP behandelt und ihre Daten in dem Register erfasst. Die Gesamtzahl an Kindern, die auf die Entwicklung einer ROP gescreent wurden, konnte retrospektiv nur für die Jahre 2006 bis 2016 eruiert werden. In diesem Zeitraum wurden 864 Kinder in der MHH gescreent, von denen 35 Kinder behandelt wurden (weitere 7 Kinder wurden in diesen Jahren an der MHH behandelt, jedoch extern gescreent und damit für die Berechnung der Inzidenz ausgeklammert, da die Gesamtzahl der Screeningfälle für die externen Kinder nicht bekannt ist). Damit beträgt die Inzidenz der behandlungsbedürftigen ROP unter den Screeningfällen an der MHH für diesen Zeitraum 4,1 %. Für den gesamten Beobachtungszeitraum betrug das mittlere Gestationsalter (GA) 25,7 Schwangerschaftswochen (SSW) (SA = 1,8; min 22,7; max 30,1), das mittlere Geburtsgewicht (GG) lag bei 763 g (SA = 235; min 400; max 1510). Von 65 Kindern waren 23 weiblich, 33 Kinder kamen nicht in der MHH auf die Welt. Von den behandelten Kindern wurden 11 (16,9 %) extern gescreent und zur ROP-Behandlung an die MHH zugewiesen, 22 Kinder (33,8 %) wurden extern geboren, zeitnah verlegt und an der MHH auf die Entwicklung einer ROP gescreent. Zwei der behandelten Kinder verstarben im Verlauf der Beobachtung, als Todesursache wurde bei beiden eine schwere chronische Lungenerkrankung dokumentiert. Ein zeitlicher oder kausaler Zusammenhang mit der ROP-Laserbehandlung bestand nach Einschätzung der behandelnden Ärzte nicht (3,5 Monate/5 Monate nach ROP-Behandlung).

**Figure 1:**
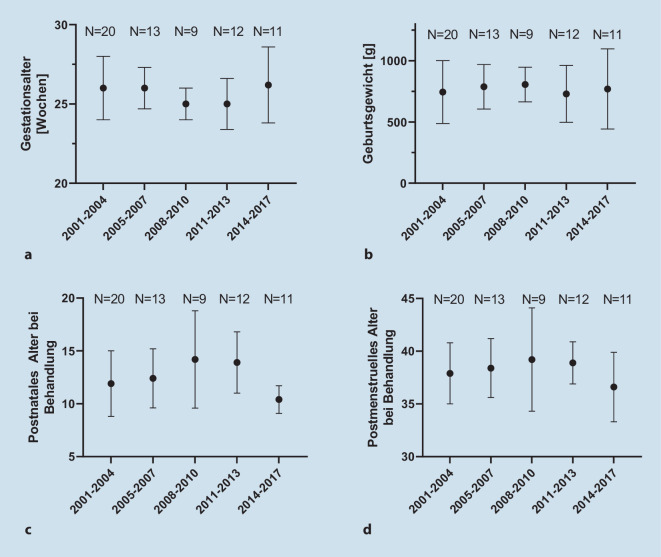

Eine Zusammenstellung der demografischen Parameter ist in Tab. 1 zu sehen. Betrachtet über die Zeit (in 5 Zeiträumen von jeweils 3 bzw. 4 Jahren), wurden keine signifikanten Veränderungen in Bezug auf das Geburtsgewicht, das Gestationsalter oder das postnatale Alter bei Behandlung gesehen (Abb. 1).

**Table 1:** 

*Gesamtzahl Patienten*		*65*
Verteilung der Patienten im Behandlungszeitraum^a^	2002–2004	20
	2005–2007	13
	2008–2010	9
	2011–2013	12
	2014–2017	11
*Gesamtzahl behandelte Augen*		*128*
Demografie bei Geburt (*N* = 65)	Gestationsalter [MW in Schwangerschaftswochen] (SA)	25,7 (1,8)
	Geburtsgewicht [MW in g] (SA)	763 (235)
	Weiblich [*n*, Kinder] (%)	23 (35)
	Von extern verlegt [*n*, Kinder] (%)	33 (51)
	Extern geboren, ROP-Screening an der MHH	22 (34)
	Extern geboren, ROP-Screening extern	11 (17)
Behandlung	Alter [PNA; MW in Wochen] (SA) (*N* = 65)	12,4 (3,2)
	Alter [PMA; MW in Wochen] (SA) (*N* = 65)	38,2 (3,2)

*MW* Mittelwert, *SA* Standardabweichung, *PMA* postmenstruelles Alter, *PNA* postnatales Alter

^a^Betrachtet werden ein Geburtszeitraum zwischen 2001 und 2016 sowie ein Behandlungszeitraum zwischen 2002 und 2017

Auffällig sind die erheblichen Schwankungen in der Anzahl an Kindern in einem Behandlungsjahr, welche ein ROP-Screening erhalten haben (Range: 36 Kinder in 2006; 123 Kinder in 2015). Da auch die Anzahl ROP-behandelter Kinder deutlich schwankt, beobachteten wir entsprechend hohe Schwankungen in der Inzidenz je Behandlungsjahr der behandlungsbedürftigen ROP (max 6 von 36 [16,7 %] in 2006, min 2 von 123 [1,6 %] in 2015, Abb. 2).

**Figure 2:**
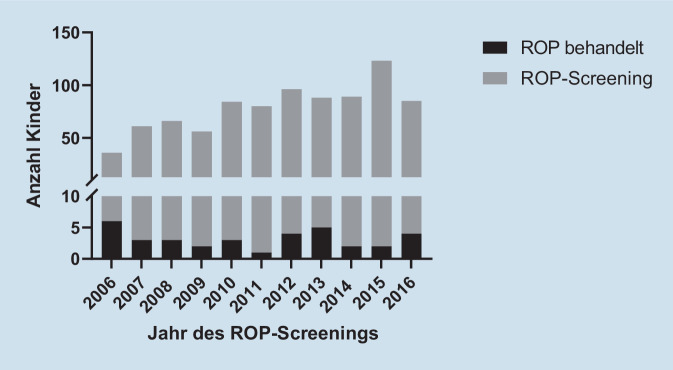

## ROP-Behandlung und Verlauf

**Figure 3:**
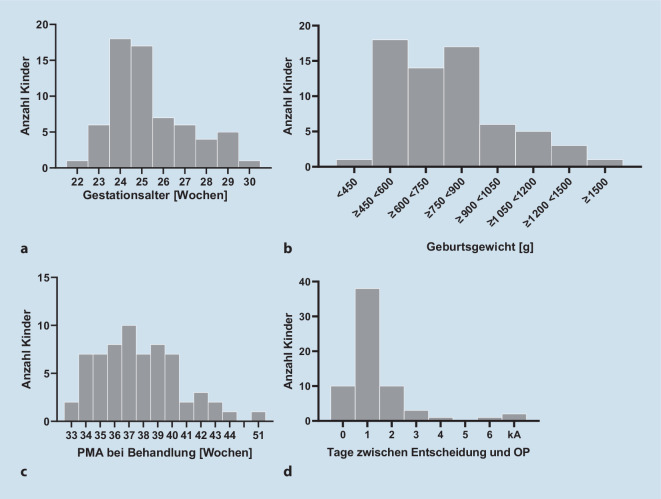

Zum Zeitpunkt der Behandlung lag das postmenstruelle Alter (PMA) der Kinder bei 38,2 Wochen (SA = 3,2; min 33,3; max 51,7), das postnatale Alter bei 12,4 Wochen (SA = 3,2; min 6,6; max 25,9). Zwischen Behandlungsentscheidung und der Behandlung vergingen im Mittel 1,2 Tage (SA = 1; min 0; max 6; 92 % innerhalb von 2 Tagen). Der Großteil der Kinder wurde mit einem PMA zwischen 33 und 40 Wochen behandelt, 4 Kinder jedoch erst spät, PMA 43, 44 bzw. 51 Wochen (Laserkoagulation, Abb. 3).

**Figure 4:**
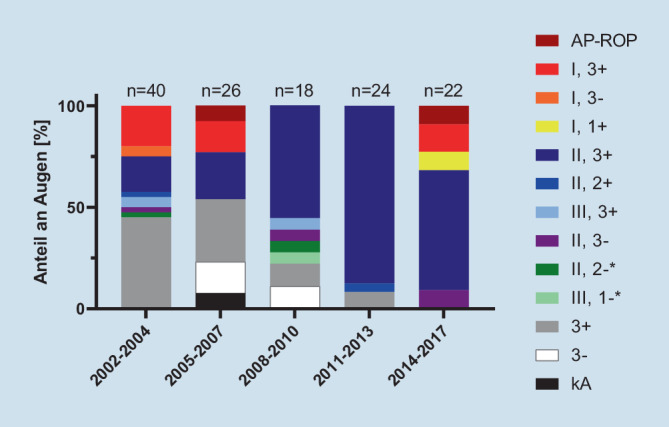

**Figure 5:**
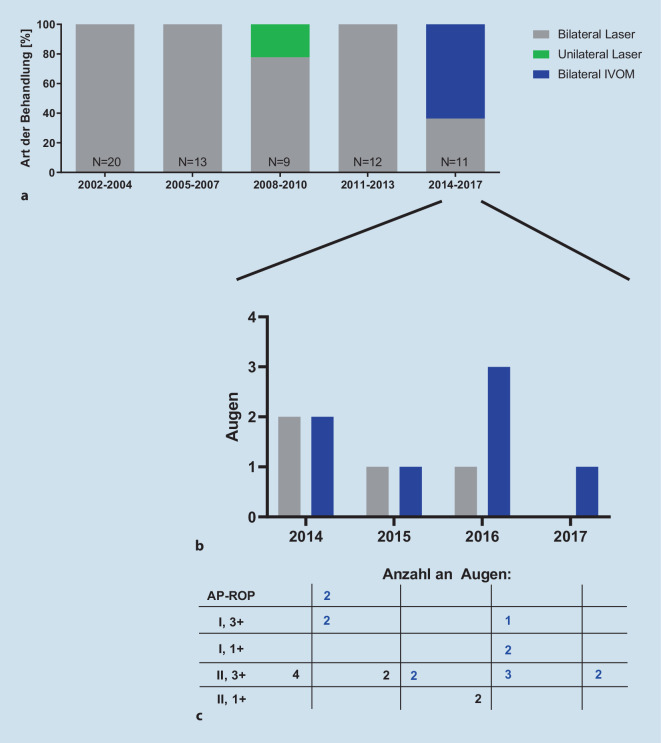

Das schwerste dokumentierte Stadium bei Behandlungsentscheidung war eine AP-ROP in 4 Augen von 2 Kindern (Tab. 2; Abb. 4). Zone-I-Erkrankungen kamen insgesamt im betrachteten Kollektiv bei 15 % aller behandelten Augen vor. Am häufigsten wurde eine Zone II, 3+ behandelt (Details s. Tab. 2; Abb. 4). Es wurden 58 Kinder mittels Laserkoagulation behandelt, davon 2 Kinder unilateral (*N* = 114 Augen, Abb. 5). Sieben Kinder wurden bilateral mittels Anti-VEGF- (0,625 mg/0,025 ml Bevacizumab) Therapie behandelt (*N* = 14 Augen). Bei 2 Kindern wurde die bilaterale Behandlung zu 2 verschiedenen Zeitpunkten durchgeführt, einmal im Abstand von 5 Tagen (Bevacizumab), einmal im Abstand von 10 Tagen (Laserkoagulation). Die Anti-VEGF-Therapie (Bevacizumab) wurde ab 2014 eingesetzt und machte im Zeitraum 2014 bis 2017 den Großteil der Behandlungen aus (Abb. 5). Kinder, die wegen AP-ROP oder einer ROP in Zone I behandelt wurden, erhielten ab 2014 immer Anti-VEGF.

**Table 2:** 

Stadium	Häufigkeit *n* (Augen)	Häufigkeit (%)
AP-ROP	4	3,1
I, 3+	15	11,5
I, 3−	2	1,5
I, 1+	2	1,5
II, 3+	57	43,8
II, 2+	2	1,5
III, 3+	3	2,3
II, 3−	4	3,1
II, 2−	2^a^	1,5
III, 1−	1^a^	0,8
Stadium 3+, ohne Angabe der Zone	30	23,1
Stadium 3−, ohne Angabe der Zone	6	4,6
Keine Angabe	2	1,5
Gesamt	130 Augen	–

^a^128 von 130 Augen wurden behandelt, 1 Auge mit Stadium II, 2− und 1 Auge mit III, 1− wurden nicht behandelt

Eine Reaktivierung einer ROP bis zu einem Schweregrad, der eine erneute Behandlungsindikation darstellt, kann sowohl nach Laserkoagulation als auch nach Anti-VEGF-Therapie auftreten. Es ist wichtig, jede Reaktivierung durch regelmäßige Nachuntersuchungen rechtzeitig zu erkennen. Im untersuchten Kollektiv wurde keine behandlungsbedürftige Reaktivierung nach Anti-VEGF-Behandlung (Bevacizumab) beobachtet. Nach Laserkoagulation kam es bei 2 Kindern zu einer Amotio (vor der ersten Behandlung lag in beiden Fällen eine ROP in Stadium 3 mit Plus Disease vor, ohne Angabe der Zone). Bei einem Kind (2003) erfolgten ROP-Screening und -Behandlung intern, es traten 6 Wochen nach Laserbehandlung (beidseits ROP Stadium 3+) am rechten Auge eine Amotio (ROP Stadium 4) auf sowie beidseits retinale Blutungen. Nach erneuter Behandlung mit Laserkoagulation links und Vitrektomie rechts weist der letzte Bericht 4 Wochen postoperativ beidseits den Befund als „stabil“ aus. Ein Kind wurde 2011 nach Screening extern zur ROP-Behandlung (beidseits) an die MHH verlegt und entwickelte trotz der Laserbehandlung am rechten Auge nach 8 Tagen eine Amotio retinae (Stadium 4b), am linken zeigte sich ein Rückgang des ROP-Stadiums. Die Erfolgsaussichten einer Operation wurden als sehr gering beurteilt und die Schwere der Gesamtbeeinträchtigung des Kindes sowie das Risiko eines Eingriffs in Allgemeinanästhesie als sehr hoch (u. a. intraventrikuläre Hämorrhagie, posthämorrhagischer Hydrozephalus, Porenzephalie, Anämie).

## Neonatologische Parameter

Neonatologische Parameter stellen neben den demografischen Parametern wichtige Faktoren bei der Entstehung einer behandlungsbedürftigen Frühgeborenenretinopathie dar. Alle Kinder mit auswertbarer neonatologischer Dokumentation (*N* = 64) erhielten zwischen der Geburt und der initialen ROP-Behandlung Sauerstoff, in 94 % (60 von 64 Kinder) lag eine invasive Beatmung (Intubation oder CPAP) vor; 92 % (59 von 64) der Kinder hatten eine bronchopulmonale Dysplasie (BPD; Stadium: 27 mild, 19 moderat, 11 schwer, 2 nicht dokumentiert). Ein persistierender Ductus arteriosus wurde in 72 % (46 der 64 Kinder) diagnostiziert, 43 Kinder erhielten eine medikamentöse Behandlung, 2 eine zusätzliche chirurgische Behandlung; 59 von 65 Kindern (91 %) benötigten Transfusionen von Erythrozytenkonzentraten, und bei 7 von 28 Kindern lag eine systemische Pilzinfektion vor (als Pilzinfektionen gewertet, wenn die Information über eine systemische Gabe von Antimykotika vorlag). Eine intraventrikuläre Hirnblutung lag bei 51 % (32 von 63) der Kinder vor, ein therapiebedürftiger Hydrozephalus bei 11 % (7 von 63 Kindern) und eine nekrotisierende Enterokolitis bei 19 % (12 von 64 Kindern).

## Diskussion

Hier wird die erste Auswertung von Daten eines einzelnen Zentrums im Rahmen des Retina.net ROP-Registers vorgestellt. Die große Fallzahl von 65 Patienten wurde durch die systematische Erfassung von Patienten über einen langen Zeitraum erreicht, die detaillierte Dokumentation der neonatologischen Parameter durch die enge Kooperation mit den Kinderärzten. Eine monozentrische Auswertung innerhalb des Retina.net ROP-Registers auf Basis der systematischen Datenerfassung in der Datenbank stellt damit eine weitere mögliche und sinnvolle Option für alle teilnehmenden Zentren dar.

Über 15 Jahre zeigten sich weitgehend unveränderte epidemiologische Parameter bei 65 Kindern mit behandlungsbedürftiger ROP und ein Therapiewechsel von Laserkoagulation zu Anti-VEGF (ab 2014). Dabei muss berücksichtigt werden, dass in den jeweils betrachteten Zeiträumen nur eine geringe Anzahl an Kindern betrachtet wurde. Kinder mit behandlungsbedürftiger ROP zeigten innerhalb der ohnehin vulnerablen Gruppe der Very-low-birthweight(VLBW)-Kinder häufig neonatologische Komorbiditäten, in > 90 % Beatmung, bronchopulmonale Dysplasie und Transfusionen.

Ein Vergleich der Parameter an dem einzelnen Zentrum mit Daten aus anderen Registern zeigt weitgehend ähnliche Resultate.

Die Inzidenz der behandlungsbedürftigen ROP innerhalb der Risikogruppe, also derjenigen Frühgeborenen, die in das ROP-Screening aufgenommen wurden, lag für den Zeitraum 2006 bis 2016 etwas höher als im Retina.net ROP-Gesamtregister (4,1 % vs. 3,2 % für die Jahre 2011 bis 2013) [18], etwas niedriger im Vergleich zum SWEDROP-Register (5,2 %, 2008 bis 2012) [5] oder dem German Neonatal Network (GNN; 4,8 %, 2009 bis 2014) (persönliche Kommunikation mit Prof. Göpel, Leiter GNN). Sie lag etwas höher als in der zahlenmäßig größten Kohorte aus einem einzelnen deutschen Behandlungszentrum (3,5 %, 1222 Kinder, 2001 bis 2007), die bislang publiziert wurde [10]. Betrachtet man die einzelnen Jahre, treten erhebliche Schwankungen im MHH-Kollektiv auf (1,6–16,7 %), die im GNN geringer ausfallen (3,9 % in 2017, 5,2 % in 2015) (persönliche Kommunikation mit Prof. Göpel, Leiter GNN). Die bisher größte deutsche Kohorte mit über 52.000 Kindern zeigt eine Inzidenz von 2,9 % [8]. Eine kleinere Studie aus Bonn/Freiburg zeigte eine Inzidenz von 2,5 % [7]. In der Schweiz liegt die Inzidenz bei 1,2 % [3]. Generell muss bei Betrachtung der Inzidenz der Frühgeborenenretinopathie berücksichtigt werden, dass die einzelnen Angaben sich häufig nicht auf exakt das gleiche Kollektiv beziehen, so werden im SWEDROP nur gescreente Kinder bis GA 30 Wochen eingeschlossen, während in unserer Auswertung alle Kinder, die sich im Screening befanden, gewertet wurden. Es muss auch berücksichtigt werden, dass sich die Kriterien in den nationalen und internationalen Empfehlungen zum ROP-Screening über den Beobachtungszeitraum mehrfach verändert haben [9, 11].

Das mittlere GA (25,7 SSW) lag im Vergleich zur BEAT-ROP- (24,2–24,5) [13], CARE-ROP- (24,6–24,7) [17] Studie sowie zum SWEDROP-Register (24,5) [5] etwas höher, vergleichbar mit dem Retina.net ROP-Gesamtregister (25,0 SSW) [19]. Analog dazu war auch das Geburtsgewicht etwas höher als in den genannten Studien. Die Tatsache, dass kaum Änderungen dieser Parameter über 15 Jahre zu sehen waren, spricht für eine sehr stabile neonatologische Betreuung an der MHH bzw. im Einzugsbereich. Auch im Retina.net-Gesamtregister wurden keine Änderungen über die Zeit (5 Jahre; 2011 bis 2015) gefunden [19], während im SWEDROP-Register im Verlauf immer jüngere und leichtere Kinder aufgrund einer ROP behandelt wurden (2008 bis 2017) [4].

In diesem hochselektiven Kollektiv der Frühgeborenen mit behandlungsbedürftiger ROP innerhalb der ohnehin schon vulnerablen Risikopopulation der VLBW-Kinder wurden > 90 % der Kinder beatmet, mit Sauerstoff versorgt oder hatten eine bronchopulmonale Dysplasie. Die nekrotisierende Enterokolitis (NEC, 12 von 64 Kindern [19 %]), aber auch intraventrikuläre Hirnblutungen (32 von 63 Kindern, 51 %) traten in der betrachteten Kohorte sehr häufig auf (vs. GNN: NEC 2,7 %) [14].

Die häufigste Behandlungsindikation an der MHH, im Gesamtregister und anderen Studien ist eine Zone II, 3+-Erkrankung (57 Augen; 44 %) [13, 17]. Eine AP-ROP (3 %) ist selten (Gesamtregister 6,2 % [19]), ein Stadium 4 wurde bei initialer Behandlung nicht beschrieben im Gegensatz zum Gesamtregister (1,7 %). Jedoch schritt bei 2 Kindern der Befund nach Laserkoagulation bis zu Stadium 4 (3 Augen) fort, was auch im Gesamtregister beschrieben wurde [18]. Dabei bestand ein Unterschied zwischen einer Reaktivierung 6 Wochen nach der Laserbehandlung und der Verschlechterung innerhalb von 8 Tagen nach Laserbehandlung. Solche Fälle machen deutlich, wie wichtig das ROP-Screening für eine frühzeitige Diagnose eines behandlungsbedürftigen Stadiums ist – sowohl initial als auch nach Laserkoagulation bzw. Anti-VEGF-Therapie [1, 11]. Insbesondere nach Anti-VEGF können diese Nachkontrollen Familien und Untersucher vor ganz besondere Herausforderungen stellen, da ein Wiederaufflammen einer aktiven behandlungsbedürftigen ROP bis zu 69 Wochen nach Erstbehandlung beschrieben ist [6, 12, 15]. Die enge Kooperation mit den betreuenden Neonatologen, Kinderärzten, Augenärzten und den Familien ist komplex, aber entscheidend für den Therapieerfolg. Zur Unterstützung des Informationsaustauschs kann ein ROP-Pass in das gelbe U‑Heft eingelegt werden [2]. Diese Komplexität zeigt sich auch in der Tatsache, dass in wenigen Fällen ungewöhnlich spät per Laserkoagulation behandelt wurde, die Partneraugen zweizeitig versorgt wurden oder Augen mit einem ROP-Stadium behandelt wurden, die im engeren Sinne der Screening- und Therapieempfehlungen noch nicht hätten behandelt werden müssen. In die Therapieentscheidung gehen bei jedem Individuum neben dem Netzhautbefund die Dynamik aus den letzten Untersuchungen ein, der Allgemeinzustand des Frühgeborenen, die neonatologische Beurteilung und die Narkosefähigkeit sowie die Vor- und Nachteile der Anti-VEGF-Therapie vs. Lasertherapie. Erfreulicherweise wurde im MHH-Kollektiv nach Anti-VEGF-Gabe kein Rezidiv einer behandlungsbedürftigen ROP beobachtet, während dieses im Gesamtregister bei 23 % der mit Anti-VEGF behandelten Patienten dokumentiert wurde [19]. Die Anti-VEGF-Therapie wurde in Hannover im Vergleich zum Gesamtdatensatz (2011) später eingeführt (2014). Im Jahr der Einführung wurde sie an der MHH bereits bei 50 % der behandelten Kinder eingesetzt, vergleichbar zum Gesamtregister im selben Jahr und seit 2016 stellt die Anti-VEGF-Behandlung die vorwiegende Behandlungsoption an der MHH dar.

## Fazit für die Praxis

- Die behandlungsbedürftige ROP ist selten. Das Retina.net ROP-Register ermöglicht die Analyse der Daten für alle teilnehmenden Zentren.
- Über 15 Jahre zeigten sich die demografischen Parameter weitgehend stabil bei 65 Kindern mit behandlungsbedürftiger ROP und ein Therapiewechsel von Laserkoagulation zu Anti-VEGF.
- ROP-Screening-Untersuchungen sind essenziell zur frühzeitigen Detektion eines behandlungsbedürftigen ROP-Stadiums sowohl initial als auch nach Therapie.
- Ein Wiederaufflammen der ROP kann insbesondere nach Anti-VEGF sehr spät auftreten.
- Die enge Kooperation mit der Neonatologie ist wichtig.
- Kinder mit behandlungsbedürftiger ROP zeigen häufig neonatologische Komorbiditäten, in > 90 % Beatmung, bronchopulmonale Dysplasie und Transfusionen.
